# A new tree species from seasonally dry tropical forest in southern Ecuador, *Spirotheca zapotillana* sp. nov. (Malvaceae), resolves a putatively disjunct distribution

**DOI:** 10.3897/phytokeys.265.162409

**Published:** 2025-10-31

**Authors:** Jorge Armijos-Barros, Diego González-Sánchez, Darío Nole-Nole, Lars Markesteijn, Adrián Escudero, Andrea Jara-Guerrero, Carlos Iván Espinosa

**Affiliations:** 1 EcoSs Lab, HUTPL Herbarium, Department of Biological and Agricultural Sciences, Universidad Técnica Particular de Loja, Loja, Ecuador Universidad Técnica Particular de Loja Loja Ecuador; 2 Doctoral Program in Natural Resources Conservation. International Doctoral School, Universidad Rey Juan Carlos, 28933 Móstoles, Madrid, Spain Universidad Rey Juan Carlos Madrid Spain; 3 Global Change Research Institute (IICG-URJC), Area of Biodiversity and Conservation, Department of Biology and Geography, Physics and Inorganic Chemistry, ESCET, Universidad Rey Juan Carlos, Mostoles, Madrid, Spain Bangor University Bangor United Kingdom; 4 School of Environmental and Natural Sciences, Bangor University, Bangor, Gwynedd, LL57 2DG, UK Universidad Rey Juan Carlos, Mostoles Madrid Spain

**Keywords:** Bombacoideae, disjunction, endemism, Equatorial Pacific, plant taxonomy

## Abstract

A new species of Malvaceae, *Spirotheca zapotillana*, is described from the Equatorial Pacific region of South America. The species occurs in seasonally dry tropical forests of El Oro and Loja provinces in southern Ecuador. It is characterized by a strictly tree-like habit, elliptical leaflets with acute apices, glandular floral receptacles, glabrous styles, oblong capsules, and reniform seeds. A morphological description of the new species is provided, along with data on its habitat and distribution, an assessment of its conservation status, and an updated key for the genus.

## ﻿Introduction

The genus *Spirotheca* Ulbr. (Malvaceae, Bombacoideae) was established in 1914 to accommodate *Spirotheca rivieri* (Decne.) Ulbr., previously classified in *Ceiba* Mill., and simultaneously to describe a new species, *Spirotheca salmonea* Ulbr. When examining a specimen collected by Weberbauer in southern Peru, Ulbrich (1914) noted similarities to *Ceiba rivieri* (Decne.) K.Schum., a species restricted to northeastern Brazil. However, a detailed morphological analysis revealed marked differences from other *Ceiba* species, including a truncated calyx, variations in petal pubescence, four pairs of thecae at the apex of each staminal lobe, absence of appendages at the base of the staminal tube, and anthers spiraled around the staminal tube before anthesis. These observations led Ulbrich to conclude that *C. rivieri* did not belong in the genus *Ceiba*. He proposed *Spirotheca*, designating *S. salmonea* Ulbr., based on the Weberbauer collection, as the type species, and transferred *Eriodendron rivieri* Decne., the basionym of *C. rivieri*, to *Spirotheca*.

Species of *Spirotheca* are morphologically distinctive, particularly in their androecium. Each flower bears five complex structures at the apex of the staminal column (here referred to as “stamens”). Each stamen comprises a group of four thecae, arranged in two superposed pairs, borne on a filament-like lobe. Furthermore, the stamens are tightly spiraled around the staminal tube from the bud stage until anthesis. Many species also exhibit a hemiepiphytic growth habit and sometimes act as stranglers that envelop and eventually kill the host tree (Gibbs and Alverson 2006; Duarte et al. 2007).

Since Ulbrich’s original description, additional species previously included in *Ceiba* have been transferred to *Spirotheca* (Cuatrecasas 1954; Gibbs and Alverson 2006), and another was described by Fernández-Alonso (2001). The taxonomic revision by Gibbs and Alverson (2006) recognized five species, all in humid neotropical forests: *S. awadendron* Fern.Alonso; *S. mahechae* Fern.Alonso; *S. michaeli* Cuatrec.; *S. rivieri* (Decne.) Ulbr. (including S. rivieri var. passifloroides [Cuatrec.] P.E.Gibbs & W.S.Alverson); and *S. rosea* (Seem.) P.E.Gibbs & W.S.Alverson. Subsequently, *S. elegans* Carv.-Sobr., M.Machado & L.P.Queiroz was described from the Caatinga region in Bahia, Brazil, representing the first record of a *Spirotheca* species from a seasonally dry tropical forest (Carvalho-Sobrinho et al. 2012) and expanding the genus to six species.

During ecological surveys in Zapotillo Cantón, Loja Province, southern Ecuador, the presence of deciduous trees with grayish trunks covered in prickles was frequently observed. These trees have often been misidentified as *Ceiba insignis* (Kunth) P.E.Gibbs & Semir, likely because they are leafless for most of the year due to seasonal rainfall. In August 2024, populations were discovered in bloom, and flowering specimens revealed floral features characteristic of *Spirotheca*. Subsequent collections and comparisons with herbarium material confirmed that these trees represent a previously undescribed species.

## Materials and methods

**Figure 1.**
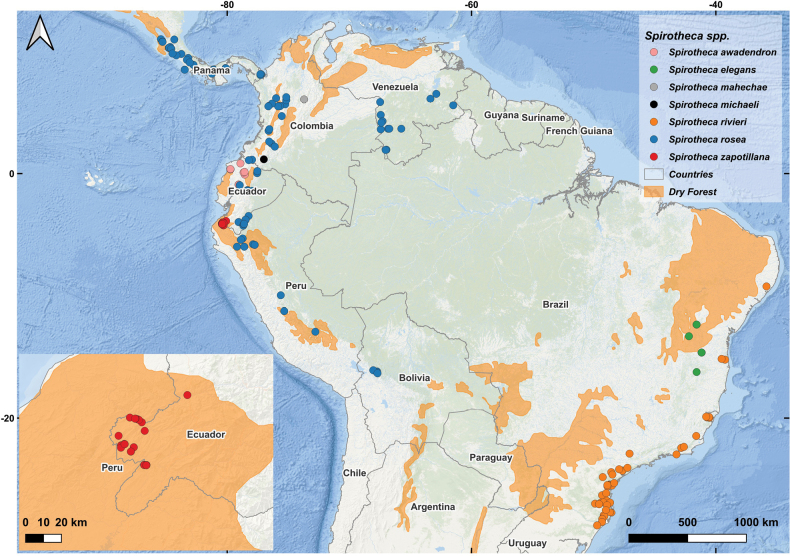
Geographic distribution of *Spirotheca*. The species most proximate in geographic range to *Spirotheca zapotillana* are *S. awadendron* and *S. rosea*. In contrast, *S. elegans* and *S. rivieri* show the greatest morphological similarity to *S. zapotillana* but are at least 4100 km distant from it.

Fieldwork was conducted in seasonally dry tropical forests of Zapotillo Cantón, Loja Province, southern Ecuador, at elevations from 400 to 900 m (Fig. 1). This area lies within the Equatorial Pacific region (Peralvo et al. 2007), which hosts some of the largest remaining patches of seasonally dry tropical forest (SDTF) on the western Andean slopes and exhibits high levels of plant endemism (Linares-Palomino et al. 2010; Espinosa et al. 2012). The climate is tropical, with an average annual temperature of 25.6 °C. Rainfall is highly seasonal; approximately 80% of annual precipitation occurs over 4 months, followed by a dry season of 6–9 months during which monthly rainfall drops below 10 mm (Maldonado and Numa 2002; Espinosa et al. 2012). Precipitation varies with elevation, from less than 500 mm annually at lower altitudes to 500–1000 mm annually at higher altitudes, yet remains strongly seasonal (Maldonado and Numa 2002).

Herbaria HUTPL, LOJA, and NY (acronyms following Thiers 2025) were consulted to verify the presence of *Spirotheca zapotillana*; only a single specimen attributable to this species was found at NY. Morphological data, including measurements of trunks, leaves, flowers, and fruits, were obtained from material collected in the field. Observations and measurements were made using a STEMI 508 stereomicroscope, and descriptive terminology follows that of Gibbs and Alverson (2006). Specimens were photographed for the preparation of a Lankester Composite Digital Plate (LCDP). Voucher specimens were deposited in the HUTPL herbarium.

Occurrence points of the new species, in conjunction with GBIF data (GBIF.org), were used to map the distribution of the genus *Spirotheca* in South America (Fig. 1, Suppl. material 1). To ensure taxonomic accuracy and reliability of the occurrence data, the following criteria were applied when selecting GBIF records: (1) the record had to be explicitly cited in protologues or taxonomic works; (2) the specimen had to be identified by a recognized specialist in Bombacoideae; and (3) the record had to include a high-quality specimen image that allowed verification of the identification. The distribution map was prepared using QGIS software.

To determine which species of *Spirotheca* are morphologically similar to *Spirotheca zapotillana*, an analysis was carried out using the approach outlined by Carvalho-Sobrinho and Queiroz (2011). The analysis was based on a matrix of discrete morphological characters compiled from species protologues (Suppl. material 2) and analyzed under a maximum parsimony framework. The character matrix was formatted as a phyDat object using the phangorn package in R (Schliep 2011), treating each trait as an unordered multi-state character. An initial tree was inferred using a Neighbor-Joining (NJ) algorithm with pairwise Hamming distances. The resulting topology was used as a starting point for tree optimization under parsimony. Branch support was assessed through a non-parametric bootstrap with 1,000 replicates.

The preliminary conservation status was assessed using the criteria recommended by the IUCN Red List (IUCN 2024). Georeferenced specimen data were imported into GeoCAT to calculate the area of occupancy (AOO) and the extent of occurrence (EOO) (Bachman et al. 2011).

## Taxonomy

### Spirotheca zapotillana

Taxon classificationPlantaeMalvalesMalvaceae

Armijos, D.P.González & Nole sp. nov.

2CBE2E60-2823-527C-A800-486729D68EB8

urn:lsid:ipni.org:names:77371267-1

**Figure 2.**
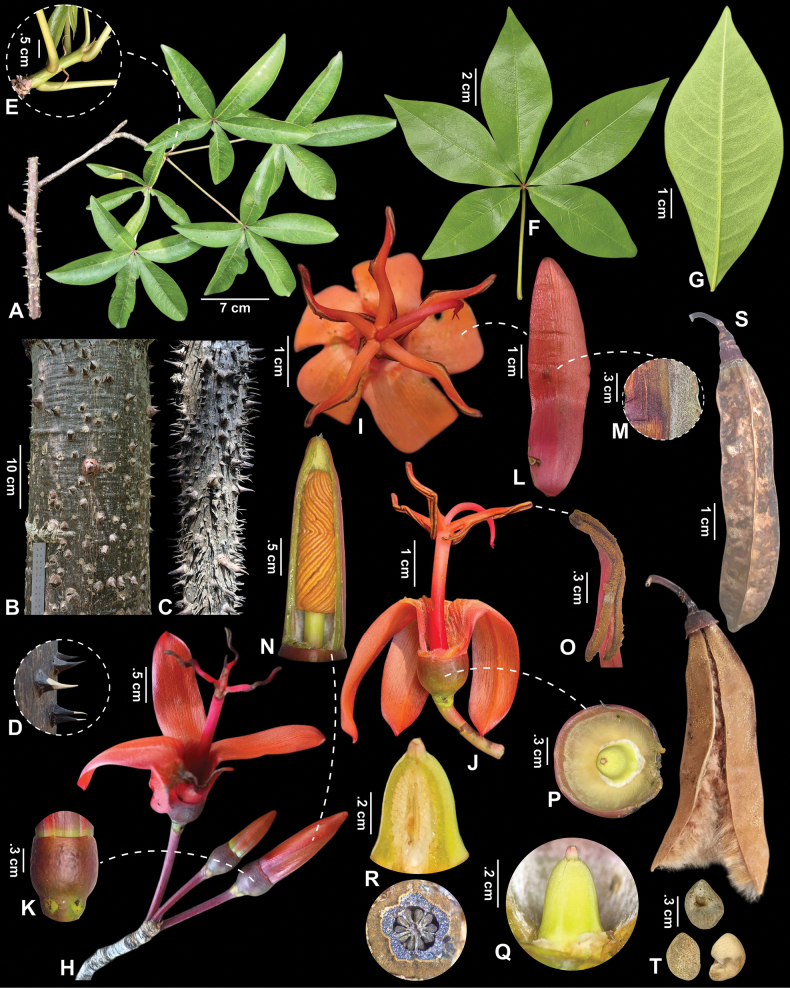
*Spirotheca zapotillana*. A. Branch with leaves; B, C. Mature and young trunk; D. Prickles; E. Cataphylls and stipules; F. Mature leaf; G. Abaxial leaflet surface; H. Cyme; I, J. Front and side views of the flower; K. Denticulate calyx and glandular receptacle; L. Petal; M. Petal with imbricate proportion pubescent (when dry); N. Arrangement of anthers prior to anthesis; O. Thecae; P. Sericeous indument inside the calyx; Q. Conical ovary; R. Longitudinal and transverse sections of the ovary (when dry); S. Fruit with ferruginous kapok; T. Seeds. Lankester Composite Digital Plate (LCDP) comprised of vouchers: A–G. Images from *J. Armijos et al. 3443*, H–Q. Images from J. *Armijos et al. 3445*, S, T. Images from *J. Armijos et al. 3444*.

Fig. 2, Suppl. material 3.

#### Diagnosis.

*Spirotheca zapotillana* Armijos, D.P.González & Nole differs from *Spirotheca elegans* Carv.-Sobr. et al. in having larger leaflets 35–90 × 15–35 mm (*vs.* 20–45 × 8–20 mm) with acute apices (*vs.* slightly retuse, obtuse, or acuminate, often mucronate), petiolules 0.9–3.8 mm long (*vs.* absent); flowers erect (*vs.* slanting downwards) and reddish (*vs.* greenish-yellow, then becoming white), staminal tubes non-articulate (*vs.* doubly articulate); capsules oblong (*vs.* obovoid or rarely spheroid), with brown (*vs.* white) kapok.

#### Type.

**Ecuador. Loja.** • Zapotillo Cantón: Parroquia Mangahurco, 4°8'30.52"S, 80°25'32.87"W, 473 m, 15 Jan 2025, *J. Armijos, D. González & D. Nole 3445* (holotype: HUTPL 15354!; isotype: LOJA!; QCNE!).

#### Description.

***Tree*** up to 15 m tall, typically found in clumps, some individuals producing clonal offshoots originating from the roots of mature trees, leafless during flowering. ***Trunks*** erect, densely covered by conical prickles, 8–33 mm long, 8–23 mm diam. at the base; bark grayish with pale, longitudinal striations; twigs with prickles. Terminal shoots covered by broadly triangular, concave cataphylls. ***Leaves*** palmately compound, clustered at branch apices. ***Petioles*** 50–100 mm long, 1–1.9 mm diam., slightly pulvinate. ***Leaflets*** (5–)7 per leaf, 35–90 × 15–35 mm, elliptical, bases cuneate, margins entire, apices acute; petiolules 0.9–3.8 mm long; adaxial surface glabrous and lustrous; abaxial surface glabrous with sparsely pubescent midrib, secondary veins prominent. ***Stipules*** linear, caducous, 4.5–12 × 0.9–1.2 mm. ***Cymes*** bearing 3–5 flowers, terminal. ***Flower buds*** 24–40 × 6–9.5 mm, linear-oblong, green to red prior to anthesis, anthers spirally arranged within the flower buds. ***Flowers*** with erect pedicels 13.5–26.1 mm long; receptacles with 4–5 small, conspicuous glands at the base. ***Calyces*** 3.5–8 × 6.5–11.2 mm, cupuliform, glabrous externally, sericeous internally, conspicuously 5-apiculate. ***Petals*** 44–52 × 9.5–12 mm, fleshy, oblong, longitudinally asymmetric, reflexed, entirely bright red, densely pubescent internally, externally pubescent on the overlapped portion of petals during the bud stage, sparsely pubescent on the non-overlapped portion. ***Staminal tube***s 25–32 × 2.5–3.4 mm, of uniform width, not articulate, sparsely covered with scaly trichomes; at apex divided into 5 free, reddish filament-like lobes, each 6–9 mm long. ***Anthers*** with two pairs of bisporangiate thecae joined at their ends, 14–18 mm long, extrorse, five in number; upper thecae 7–9 mm long; lower thecae 5–8 mm long. ***Ovaries*** 5.7 × 2.8 mm, greenish, conical, densely pubescent, 5-locular. ***Styles*** 14–20.5 mm long, slender, declinate at anthesis; stigmas 5-branched. ***Capsules*** 65–80 × 13–15.5 mm, 5-ribbed, 5-valved, oblong, apex rounded, containing abundant brown kapok. ***Seeds*** 5 × 3.5 mm when mature, reniform, brown.

#### Additional specimens examined.

**Ecuador. El Oro**: • [Las Lajas cantón], Puyango and vicinity, 300–900 m elev., Agu. 1978, *D.C. Daly 71* (NY [05154227 as image!]). **Loja**: • Zapotillo cantón, La Manga, 4°13'42.54"S, 80°18'31.23"W, 479 m elev., 16 May 2025, *J.L. Armijos Barros et al. 3443* (HUTPL [15352]!); • Ibid., 4°13'44.38"S, 80°17'51.13"W, 473 m elev., 16 May 2025, *J.L. Armijos Barros et al. 3444* (HUTPL [15353]!); • Mangahurco, 4°7'49.11"S, 80°25'30.87"W, 496 m elev., 16 May 2025, *J.L. Armijos Barros et al. 3446* (HUTPL [15355]!); • Baño del Inca, 4°7'25.83"S, 80°24'26.98"W, 463 m elev., *16 May* 2025, *J.L. Armijos Barros et al. 3447* (HUTPL [15356]!); • Ibid., 4°7'26.33"S, 80°24'26.87"W, 464 m elev., 16 May 2025, *J.L. Armijos Barros et al.* 3448 (HUTPL [15357]!).

#### Etymology.

The specific epithet refers to Zapotillo Cantón, southern Ecuador. We chose this name because the majority of documented populations of *Spirotheca zapotillana* occur in this area. In addition, this area comprises one of the last intact remnants of Ecuador’s seasonally dry tropical forest habitat, a highly threatened ecosystem vulnerable to degradation and fragmentation.

#### Distribution and habitat.

**Figure 3.**
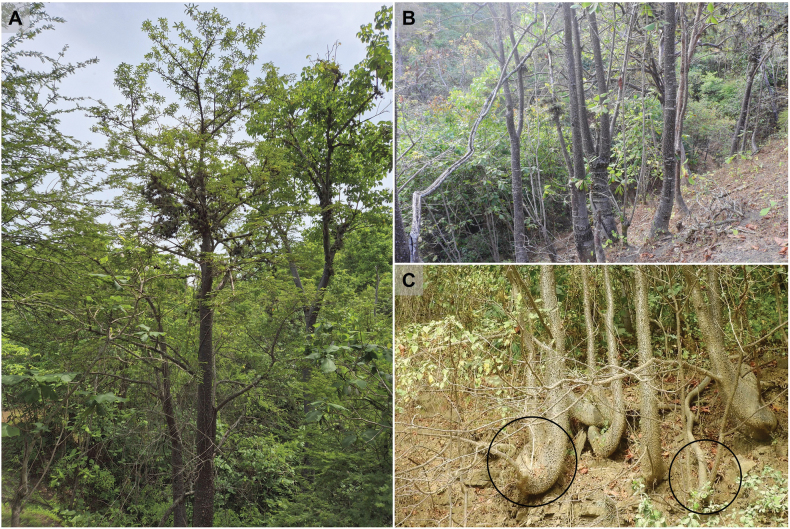
*Spirotheca zapotillana*. A, B. Individuals observed growing in clumps at the La Manga locality (wet season); C. Individuals associated with three or more clonal offshoots at the Mangahurco locality (dry season).

The species is currently known from four populations within the SDTF of Zapotillo Cantón of Loja Province (at 450–600 m elevations) and one unstudied population in Las Lajas Cantón of El Oro Province (somewhere between 300 and 900 m). Each of the Zapotillo populations contains an average of seven individuals. Mature individuals grow in clumps; some are typically associated with three or more clonal offshoots. These populations occur on rocky hillsides, across a relatively extensive area in deciduous and semi-deciduous forest types characterized by pronounced seasonality (Fig. 3).

#### Phenology.

*Spirotheca zapotillana* produces leaves at the onset of the rainy season (from January to May). Leaf senescence occurs at the end of the rainy period, and bud formation and flowering begin between June and July. Flowers are present from July to October, and fruiting occurs from September to October.

#### Preliminary conservation status.

Considering the populations reported in this study, the estimated area of occupancy (AOO) of *Spirotheca zapotillana* is restricted to 52 km^2^, and its extent of occurrence (EOO) is approximately 660 km^2^. Based on these metrics, *S. zapotillana* qualifies as Endangered (EN) under the IUCN Red List criteria B1ab(iii,v)+2ab(iii,v) (IUCN 2024). However, it is likely that additional populations occur in unsurveyed areas of tropical dry forests in the Equatorial Pacific region, including across the Peruvian border; thus, the species’ full distribution range may be more extensive than currently known.

The populations identified to date are located in areas subject to intense livestock pressure. Notably, no seedlings or saplings of *Spirotheca zapotillana* were recorded during monitoring conducted between 2022 and 2025. These results highlight the need for urgent conservation measures, including (1) expanded surveys to determine the species’ actual distribution and (2) the immediate implementation of conservation strategies for the known populations.

#### Discussion.

**Figure 4.**
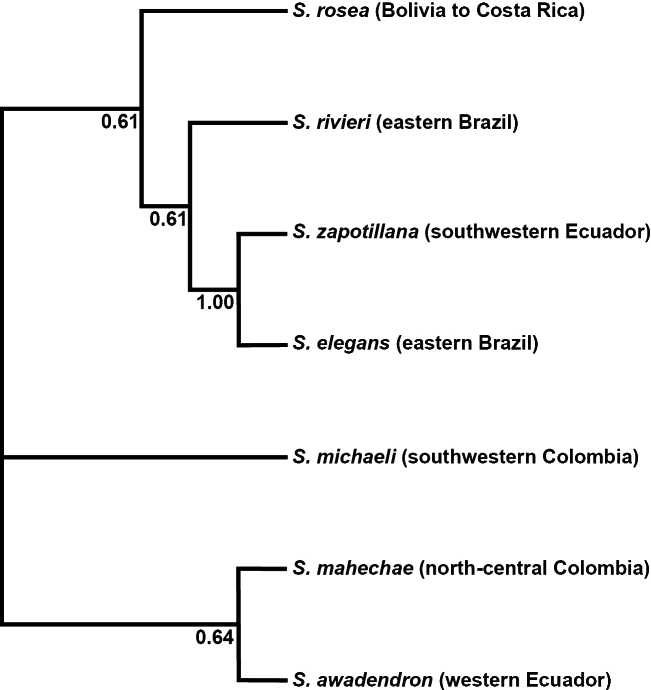
Maximum parsimony tree based on morphological characters of *Spirotheca* species. Bootstrap support values (1000 replicates) are shown below the branches. The analysis was performed using unordered multistate characters; the tree was inferred from a neighbor-joining starting topology and optimized under a parsimony criterion. Species distribution ranges are given in parentheses.

The strict consensus tree resulting from maximum parsimony analysis of morphological characters (Fig. 4) suggests that *Spirotheca zapotillana* is sister to *S. elegans*, with high bootstrap support (BS=1.0) due to the strong morphological similarity of these two species. This clade was nested within a moderately supported group (BS=0.67) that also included *S. rivieri*.

*S. zapotillana* differs from *S. elegans* (Table 1) in having cymes of 3–5 flowers (*vs.* 1–3 flowers). The petals are uniformly reddish (*vs.* initially greenish-yellow, later turning white), with the inner surface densely pubescent and the outer surface pubescent only on the overlapped portion (*vs.* minutely and densely pubescent on both surfaces). The staminal tube is cylindrical, with scaly trichomes (*vs.* doubly swollen with tufted trichomes, becoming glabrous at the apex). The fruit is an oblong capsule (*vs.* obovoid or spheroid) that contains brown kapok (*vs.* white kapok) and reniform seeds (*vs.* pyriform).

**Table 1.** Comparison of *Spirotheca zapotillana* to morphologically similar species.

Trait	*S. zapotillana*	*S. elegans* (Carvalho-Sobrinho et al. 2012)	*S. rivieri* (Gibbs and Alverson 2006)
Habit	Tree up to 15 m	Tree up to 12 m	Epiphytic shrubs or trees 20–30 m after strangling the host
Leaflets	5(–7)	(3–)4–5	3–7
	35–90 × 15–35 mm, glabrous, elliptical, apex acute	20–45 × 8–20 mm, glabrous, oblong to narrowly obovate, slightly conduplicate, apex slightly retuse, obtuse, or acuminate, often mucronate	30–80 × 8–30 mm, glabrous, elliptic-oblong to elliptic-obovate, apex obtuse and emarginate
Petiolules	0.9–3.8 mm long	Absent	3 mm long or subsessile
Flowers	In 3–5-flowered cymes, erect, pedicels 13.5–26.1 mm long, receptacle with 4–5 glands	In 1–3(-5)-flowered cymes, slanting downwards, pedicels 20–25 mm long, receptacle with 5–8 glands	Borne singly, erect, pedicels 12–24 mm long
Calyces	3.5–8 × 6.5–11.2 mm, 5–apiculate, glabrous externally	6–8 × 4–5 mm, 5–apiculate, glabrous externally	6 mm long, truncate, glabrous externally
Petals	44–52 × 9.5–12 mm, reddish, oblong, densely pubescent internally, pubescent only on the imbricate portion externally	40–45 × 7–8 mm, greenish-yellow and becoming white, linear-oblong, tufted trichomes on both surfaces	40–55 × 5–8 mm, reddish, linear-oblong, puberulent on the outside
Staminal tubes	25–32 × 2.5–3.4 mm, cylindrical and not articulate, with sparse, scaly trichomes	23–25 mm long, doubly articulate, densely covered by tufted trichomes at the base, glabrous distally	30–40 mm long, articulate or not, densely tomentose at the base, sparse distally
Ovaries	Conical, greenish, densely pubescent	Conical, densely pubescent	Densely pubescent
Styles	14–20.5 mm long, glabrous, reddish	ca. 14 mm, pinkish	Lower portion densely hairy, reddish
Capsules	65–80 × 13–15.5 mm, oblong, kapok brown	30–35 × 30–65 mm, obovoid or rarely spheroid, kapok white	90 × 55 mm, elliptic
Seeds	5 × 3.5 mm, reniform, brownish	5–6 × 6–7 mm, pyriform, brownish	

*S. zapotillana* resembles *S. rivieri* (Table 1) in having reddish flowers and a trunk covered with conical prickles. In contrast, it differs in habit, being strictly tree-like, up to 15 m tall (*vs.* hemiepiphytic shrubs with a strangler habit or trees 20–30 m tall derived from them). The leaves bear (5–)7 leaflets (*vs.* 3–7), with elliptical laminae (*vs.* elliptic-oblong to elliptic-obovate) and an acute apex (*vs.* obtuse and emarginate apex). Calyces are 5–apiculate (*vs.* truncate). The staminal tube is non-articulate (*vs.* articulate or not) with sparse, scaly trichomes (*vs.* densely tomentose at the base, sparse distally). The style is glabrous (*vs.* densely hairy). Capsules are oblong, 65–80 mm long (*vs.* elliptic, up to 90 mm).

In 1978, a leafless specimen of *Spirotheca* (*Daly 71*) was collected from the SDTF of southernmost El Oro province. Although its floral characteristics closely resembled those of S. rivieri var. rivieri, the location in Ecuador was strikingly disjunct from that species’ known range in southeastern Brazil. Gibbs and Alverson (2006) noted this specimen as an unresolved anomaly within the genus. Our study concludes that this mystery specimen represents *S. zapotillana*. We look forward to a molecular study that can resolve whether our new species is in fact most closely related to a species in Brazil or if the morphological similarities (Fig. 4) are due to convergence in dry forest habitats.

### Expanded Key to Species of *Spirotheca*

Based on Carvalho-Sobrinho et al. 2012.

**Table:** 

1a	Petals 40–55 mm, brilliant red or pinkish-white throughout anthesis	**2**
2a	Flowers erect; petals brilliant red; staminal tube red; anthers with upper thecae 6–9 mm and lower thecae 5–11 mm long	**3**
3a	Epiphytic shrubs with strangler habit or trees 20–30 m derived from such; leaflet apices obtuse and emarginate; staminal tubes articulate or not; styles hairy at the base; capsules elliptic	***S. rivieri***
3b	Tree up to 15 m; leaflet apices acute; staminal tubes cylindrical and not articulate; styles glabrous; capsules oblong	***S. zapotillana***
2b	Flowers slanting downwards; petals pinkish-white, asymmetrical, with a wing and a crispate margin; staminal tubes yellowish, doubly swollen; anthers with upper and lower thecae 3–4 mm long	***S. elegans***
1b	Petals 60–120 mm, initially whitish green, becoming pinkish or salmon, and finally dark reddish	**4**
4a	Staminal tubes 45–47 mm, lacking an articulation, more or less uniformly sericeous below, sparsely so above	**5**
5a	Petals ca. 120 mm; leaflets 70–160 × 30–56 mm, oblanceolate with an acuminate apex	***S. michaeli***
5b	Petals 75–85 mm; leaflets 40–50 × 18–22 mm, narrowly obovate	***S. mahechae***
4b	Staminal tube 12–45 mm, with a distinct articulation, densely grey tomentose below, variably and usually sparsely sericeous above	**6**
6a	Leaflets glabrous or glabrate, sometimes with sparse hairs or papillose or sparsely glandular-scurfy below (i.e., on the abaxial surface)	***S. rosea***
6b	Leaflets densely stellate-tomentose below	***S. awadendron***

## Supplementary Material

XML Treatment for Spirotheca zapotillana

## ﻿Additional information

### Use of AI

No use of AI was reported.

### Author ORCIDs

Jorge Armijos-Barros https://orcid.org/0000-0001-7448-8211

Diego González-Sánchez https://orcid.org/0000-0002-9464-4469

Darío Nole-Nole https://orcid.org/0009-0002-8084-5494

Lars Markesteijn https://orcid.org/0000-0003-3046-3121

Adrián Escudero https://orcid.org/0000-0002-1427-5465

Andrea Jara-Guerrero https://orcid.org/0000-0001-9394-2790

Carlos Iván Espinosa https://orcid.org/0000-0002-5330-4505

### Data availability

All of the data that support the findings of this study are available in the main text or Supplementary Information.

## Supplementary materials

Supplementary material 1 Georeferenced records used to generate the distribution map of *Spirotheca* Data type docx Explanation note The Reference column indicates the origin of each record: A GBIF.org; B. Gibbs and Alverson (2006); C records from the present study. https://binary.pensoft.net/file/1455160This dataset is made available under the Open Database License (http://opendatacommons.org/licenses/odbl/1.0/). The Open Database License (ODbL) is a license agreement intended to allow users to freely share, modify, and use this Dataset while maintaining this same freedom for others, provided that the original source and author(s) are credited.Jorge Armijos-Barros, Diego González-Sánchez, Darío Nole-Nole, Lars Markesteijn, Adrián Escudero, Andrea Jara-Guerrero, Carlos Iván Espinosa

Supplementary material 2 Data matrix for the phylogenetic analysis of *Spirotheca* Data type docx Explanation note Morphological characters utilized in the analysis were obtained from protologues (Cuatrecasas 1954; Fernández-Alonso 2001; Carvalho-Sobrinho et al. 2012) and a monographic study (Gibbs et al. 2006). Qualitative and quantitative traits were transformed into multistate characters. https://binary.pensoft.net/file/1455161This dataset is made available under the Open Database License (http://opendatacommons.org/licenses/odbl/1.0/). The Open Database License (ODbL) is a license agreement intended to allow users to freely share, modify, and use this Dataset while maintaining this same freedom for others, provided that the original source and author(s) are credited.Jorge Armijos-Barros, Diego González-Sánchez, Darío Nole-Nole, Lars Markesteijn, Adrián Escudero, Andrea Jara-Guerrero, Carlos Iván Espinosa

Supplementary material 3 Supplementary figures Data type pdf https://binary.pensoft.net/file/1455162This dataset is made available under the Open Database License (http://opendatacommons.org/licenses/odbl/1.0/). The Open Database License (ODbL) is a license agreement intended to allow users to freely share, modify, and use this Dataset while maintaining this same freedom for others, provided that the original source and author(s) are credited.Jorge Armijos-Barros, Diego González-Sánchez, Darío Nole-Nole, Lars Markesteijn, Adrián Escudero, Andrea Jara-Guerrero, Carlos Iván Espinosa
