# Supplementary material for: A new tree species from seasonally dry tropical forest in southern Ecuador, Spirotheca zapotillana sp. nov. (Malvaceae), resolves a putatively disjunct distribution
Source: PhytoKeys. 2025 Oct 31;265:181–92. doi: 10.3897/phytokeys.265.162409 (PMC12595510; doi:10.3897/phytokeys.265.162409)
Supplement: Supplementary material 1 — Georeferenced records used to generate the distribution map of Spirotheca [file phytokeys-265-181_article-162409__-s001.docx]

**Supplementary material 1.** Georeferenced records used to generate the distribution map of *Spirotheca*. The Reference column indicates the origin of each record: A GBIF.org; B. Gibbs and Alverson (2006); C records from the present study.

| Species | Country Code | Latitude | Longitude | Altitude | Event  Date | Collector | Coll. Number | Herbarium Acronym | | Reference |
| --- | --- | --- | --- | --- | --- | --- | --- | --- | --- | --- |
| *S. awadendron* | EC | 0°55'44.0"S | 79°3'21.0"S | 1610 m | 18 Jan 1949 | Neill | 17916 | ECUAMZ | | A |
| *S. awadendron* | EC | 0°54'0.0"S | 79°3'59.98"S | 850–1000 m | 24 Jul 1984 | Dodson et al. | 14395 | WIS | | B |
| *S. awadendron* | EC | 0°54'0.0"S | 79°3'59.98"S | 750–900 m | 19 Jul 1982 | Dodson & Embree | 13364 | MO | | B |
| *S. awadendron* | EC | 00°21'N | 79°44'W |  | 11 Mar 1998 | Clark et al. | 4642 | F | | B |
| *S. awadendron* | EC | 00°21'N | 79°44'W |  | 01 Jan 1997 | Clark et al. | 3743 | US | | B |
| *S. awadendron* | EC | 00°21'N | 79°44'W | 500 m | 04 Jan 1997 | Clark et al. | 3708 | holotype: QCNE, n.v.; isotypes: COL, n.v, MO, US | | B |
| *S. awadendron* | EC | 00°21'N | 79°44'W |  | 16 Dec 1994 | Clark et al. | 401 | MO, NY, US, WIS | | B |
| *S. awadendron* | EC | 0°49'59.99"N | 78°55'0.0"S | 500 m | 22 Jan 1901 | Rubio | 388 | LOJA | | A |
| *S. awadendron* | EC | 00°22'N | 79°44'W | 650 m | 12 Mar 1997 | Clark et al. | 4091 | F, MO, US | | B |
| *S. awadendron* | EC | 0°54'0.0"S | 79°3'59.98"S | 1300 m | 07 Aug 1984 | Dodson et al. | 15194 | WIS | | B |
| *S. awadendron* | EC | 00°05'N | 78°55'W | 500 m | 01 Jul 1990 | Rubio & Alverson | 388 | MO, WIS | | B |
| *S. awadendron* | EC | 0°7'60.0"N | 78°34'60.0"S | 1450 m | 12 Jul 1900 | Tipaz | 194 | ECUAMZ | | A |
| *S. awadendron* | EC | 0°5'31.0"N | 78°39'41.0"S | 1250 m | 03 Aug 1947 | Palacios | 17382 | MO | | A |
| *S. awadendron* | EC | 00°08'N | 78°35'W | 1200–1700 m | 20 May 1991 | Tipaz & Quelal | 194 | WIS | | B |
| *S. elegans* | BR | 12°21'03.5''S | 41°34'42.0''W | 691 m | 18 Apr 2011 | de Carvalho-Sobrinho | 3055 | holotype: HUEFS; isotypes: RB, SPF | | B |
| *S. elegans* | BR | 14°38'15.1''S | 41°10'06.5''W | 402 m | 18 Sep 2010 | de Carvalho-Sobrinho | 2868 | HUEFS | | B |
| *S. elegans* | BR | 14°38'15.1''S | 41°10'06.5''W | 402 m | 12 Mar 2011 | de Carvalho-Sobrinho | 2964 | HUEFS | | B |
| *S. elegans* | BR | 14°38'15.1''S | 41°10'06.5''W | 400 m | 20 May 2011 | de Carvalho-Sobrinho | 3094 | HUEFS | | B |
| *S. elegans* | BR | 14°38'15.1''S | 41°10'06.5''W | 400 m | 16 Jul 2011 | de Carvalho-Sobrinho | 3181 | HUEFS | | B |
| *S. elegans* | BR | 12°20'60''S | 41°34'40''W |  | 07 Feb 2008 | Venâncio & F. Esteves | 11 | ALCB | | B |
| *S. elegans* | BR | 13°18'12.8''S | 42°12'24.1''W | 668 m | 14 Mar 2011 | de Carvalho-Sobrinho | 2977 | HUEFS | | B |
| *S. elegans* | BR | 16°13'51.0"S | 41°34'34.0"S | 664 m | 16 Jun 2014 | de Paula et al. | 744 | RB | | A |
| *S. mahechae* | CO | 6°4'1.2"N | 73°42'25.2"S | 2200 m | 01 Jul 1995 | Mahecha & al. | 8572 | B |  |  |
| *S. michaeli* | CO | 01°10'N | 77°00'W | 2200–2300 m | 01 Jan 1942 | Fray Miguel | 25B | holotype: F | | B |
| *S. rivieri* | BR | 9°13'39.0"S | 35°51'29.0"S |  | 23 nov 2012 | Mota | 11837 | MAC | | A |
| *S. rivieri* | BR | 15°13'8.0"S | 39°14'53.0"S |  | 18 Dec 1968 | dos Santos | 325 | CEPEC | | A |
| *S. rivieri* | BR | 15°9'16.0"S | 39°31'52.0"S |  | 10 Dec 2004 | Amorim | 4490 | CEPEC | | A |
| *S. rivieri* | BR | 24°18'0.0"S | 47°38'60.0"S |  | 15 Dec 2002 | Breier | 785 | UEC | | A |
| *S. rivieri* | BR | 19°58'17.0"S | 40°31'49.0"S |  |  | Chamas | 382 | MBML | | A |
| *S. rivieri* | BR | 19°56'23.1"S | 40°43'55.8"S |  | 16 Jul 2003 | Assis | 958 | MBML | | A |
| *S. rivieri* | BR | 19°54'27.0"S | 40°33'11.0"S |  |  | Kollmann | 5123 | MBML | | A |
| *S. rivieri* | BR | 19°54'27.0"S | 40°33'11.0"S |  |  | Vervloet | 1564 | MBML | | A |
| *S. rivieri* | BR | 19°53'14.0"S | 40°47'45.0"S |  |  | Demuner | 1323 | MBML | | A |
| *S. rivieri* | BR | 25°25'56.94"S | 48°42'44.13"S |  | 20 jul 1972 | Hatschbach | 29808 | MBM, NY | | B |
| *S. rivieri* | BR | 25°26'60.0"S | 48°43'0.12"S | 75 m | 10 jul 1985 | Hatschbach | 49519 | ASU | | A |
| *S. rivieri* | BR | 25°26'60.0"S | 48°43'0.12"S | 75 m | 10 Jul 1985 | Hatschbach & Zelma | 4915 | MBM | | B |
| *S. rivieri* | BR | 24°49'25.0"S | 49°15'39.0"S |  | 01 Aug 1985 | Hatschbach & Souza | 49542 | MBM | | B |
| *S. rivieri* | BR | 25°28'37.0"S | 48°50'3.0"S |  | 18 jul 1981 | Hatschbach | 43950 | MBM, NY | | B |
| *S. rivieri* | BR | 25°31'13.0"S | 48°30'33.0"S |  | 19 Jun 1970 | Hatschbach | 24408 | K, NY, RFA, US | | B |
| *S. rivieri* | BR | 25°34'45.0"S | 48°52'33.0"S | 232.5 | 7 Jun 2013 | Völtz | 249 | MBM | | A |
| *S. rivieri* | BR | 25°28'40.0"S | 48°53'4.0"S | 240 m | 11 Jul 2020 | Brotto | 3887 | HCF | | A |
| *S. rivieri* | BR | 25°18'25.0"S | 48°19'44.0"S |  | 5 jul 1967 | Hatschbach & Koezieki | 16663 & 48921 | F, K, MBM, NY, US | | B |
| *S. rivieri* | BR | 22°57'57.0"S | 43°13'32.0"S |  |  | Nadruz | 3890 | RB | | A |
| *S. rivieri* | BR | 22°25'60.0"S | 42°49'60.0"S |  |  | de Lima |  | RB | | A |
| *S. rivieri* | BR | 21°27'33.0"S | 41°36'31.0"S | 270 m |  | Costa | 461 | RB | | A |
| *S. rivieri* | BR | 22°21'0.0"S | 42°36'59.98"S |  | 8 jul 1989 | Kurtz et al. | 88 | F, MO, R, UEC | | B |
| *S. rivieri* | BR | 27°58'40.0"S | 49°10'47.0"S | 400 m | 02 Aug 2020 | Külkamp | 1329 | FLOR | | A |
| *S. rivieri* | BR | 27°7'35.89"S | 48°54'36.53"S | 50 m | 28 Jul 1950 | Klein & Smith | 204 | NY | | B |
| *S. rivieri* | BR | 26°18'25.0"S | 49°18'48.0"S | 650 m | 1 Jul 2018 | Schwirkowski | 2858 | FURB | | A |
| *S. rivieri* | BR | 26°44'54.73"S | 48°56'28.24"S | 400 m | 19 Jul 1954 | Reitz | 1999 | NY | | A |
| *S. rivieri* | BR | 26°59'51.0"S | 49°53'11.0"S | 680 m | 23 Aug 2020 | Kassner-Filho | 5785 | FURB | | A |
| *S. rivieri* | BR | 27°45'9.0"S | 48°32'33.0"S | 118 m |  | Riella | 14 | FLOR | | A |
| *S. rivieri* | BR | 26°54'58.0"S | 49°25'12.0"S | 671 m | 12 Sep 2016 | Funez | 5513 | FURB | | A |
| *S. rivieri* | BR | 26°53'11.0"S | 49°11'49.0"S |  | 04 Jul 2019 | Bones | 212 | FURB | | A |
| *S. rivieri* | BR | 26°54'43.22"S | 48°39'44.14"S | 100 m | 12 Aug 1954 | Klein & Reitz | 801 | NY, R, US | | B |
| *S. rivieri* | BR | 27°7'35.89"S | 48°54'36.53"S | 50 m | 28 Jul 1950 | Klein | 204 | NY | | A |
| *S. rivieri* | BR | 27°43'9.0"S | 48°39'50.0"S | 162 m | 07 Jul 2019 | Brotto | 3702 | FLOR | | A |
| *S. rivieri* | BR | 28°4'13.0"S | 49°8'18.0"S | 200 m | 15 Jul 2011 | Carneiro | 69 | FURB | | A |
| *S. rivieri* | BR | 26°8'29.0"S | 48°58'57.0"S | 215 m |  | Bilk | 142 | JOI | | A |
| *S. rivieri* | BR | 27°2'13.0"S | 49°33'33.0"S | 229 m |  | de Gasper | 3361 | CESJ | | A |
| *S. rivieri* | BR | 27°2'13.0"S | 49°33'33.0"S | 229 m |  | Gasper | 3361 | BHCB | | A |
| *S. rivieri* | BR | 28°17'7.0"S | 49°17'56.0"S | 350 m | 27 Jul 1991 | Citadini-Zanette | 1767 | CRI | | A |
| *S. rivieri* | BR | 28°17'7.0"S | 49°17'56.0"S | 350 | 06 Aug 1993 | Waechter | 2585 | CRI | | A |
| *S. rivieri* | BR | 27°32'4.0"S | 49°0'16.0"S | 357 m | 16 jul 2019 | Kassner-Filho | 5503 | FURB | | A |
| *S. rivieri* | BR | 28°31'22.0"S | 49°19'30.0"S |  | 26 jan 2010 | Rocha |  | CRI | |  |
| *S. rivieri* | BR | 28°48'4.0"S | 49°43'18.0"S |  | 04 Jul 2019 | Biff |  | CRI | |  |
| *S. rivieri* | BR | 22°54'20.16"S | 47°3'56.7"S | 650 m | 07 Jun 2005 | Amaral | 82 | IAC | | A |
| *S. rivieri* | BR | 24°18'0.0"S | 47°38'60.0"S |  |  | Rocca | 71 | UEC | | A |
| *S. rivieri* | BR | 24°18'0.0"S | 47°38'60.0"S |  |  | Rocca | 81 | UEC | | A |
| *S. rivieri* | BR | 24°10'48.0"S | 47°55'12.0"S | 400 m |  | Lima |  | ESA | | A |
| *S. rivieri* | BR | 24°3'46.0"S | 47°14'13.0"S | 600 m | 2 jan 2008 | Souza | 36056 | ESA | | A |
| *S. rivieri* | BR | 24°25'3.7"S | 48°33'52.8"S |  |  | Melo | 7081 | HRCB | | A |
| *S. rivieri* | BR | 24°37'3.0"S | 48°28'10.0"S |  | 28 Mar 2005 | Carboni | 166 | ESA | | A |
| *S. rosea* | BO | 16°11'S | 67°44'W |  | 17 Jun 1964 | Badcock | 200 | K | | B |
| *S. rosea* | BO | 16°13'S | 67°47'W | 1800–1900 m | 16 May 1990 | Solomon et al. | 18979 | B |  |  |
| *S. rosea* | BO | 16°05'S | 68°03'W | 1800 m | 30 Jan 1988 | Solomon | 17754 | MO, WIS | | B |
| *S. rosea* | BO | 16°20'S | 67°42'W | 1730 m | 03 May 1989 | Smith & Smith | 13130A | IS, MO, SCZ, WIS | | B |
| *S. rosea* | CO | 6°9'59.98"N | 76°0'0.0"S | 1700 m |  | McPherson | 13252 | MO | | A |
| *S. rosea* | CO | 5°39'59.98"N | 75°15'0.0"S | 2200 m |  | McPherson | 13091 | MO | | A |
| *S. rosea* | CO | 5°30'59.98"N | 75°39'59.98"S | 2140 m |  | Betancur | 1057 | COL | | A |
| *S. rosea* | CO | 6°0'0.0"N | 75°10'0.0"S | 1830 m |  | Juncosa | 1387 | MO | | A |
| *S. rosea* | CO | 5°30'0.0"N | 75°49'59.99"S | 2150 m |  | McPherson | 12918 | MO | | A |
| *S. rosea* | CO | 6°15'0.0"N | 75°10'0.0"S | 1850 |  | Albert | 5110 | HUA | | A |
| *S. rosea* | CO | 02°27'N | 76°22'W | 1700 m | 11 Apr 1939 | Alston | 7964 | NY | | B |
| *S. rosea* | CO | 2°37'0.0"N | 76°34'0.0"S |  | 15 May 1968 | Patiño | 331 | BR | | A |
| *S. rosea* | CO | 5°43'15.0"N | 76°20'25.0"S | 1160 m |  | Gentry | 23716 | HUA | | A |
| *S. rosea* | CO | 8°7'4.97"N | 77°13'12.72"S | 1150 m |  | Gentry | 17035 | COL | | A |
| *S. rosea* | CO | 2°12'3.01"N | 76°6'7.94"S | 2150 m |  | Cabrera | 5110 | CUVC | | A |
| *S. rosea* | CO | 1°7'59.99"N | 77°57'59.98"S | 1750 m |  | Gentry | 35168 | COL | | A |
| *S. rosea* | CO | 4°42'10.98"N | 75°32'6.97"S | 2140 m |  | Rangel | 5742 | MO | | A |
| *S. rosea* | CO | 3°30'0.0"N | 76°37'0.0"S | 1950–2050 m | 15 Jul 1984 | Gentry et al. | 48129 | MO, WIS | | B |
| *S. rosea* | CO | 3°37'59.99"N | 76°32'60.0"S | 1875 m |  | Gentry | 65425 | MO | | A |
| *S. rosea* | CO | 5°30'0.0"N | 76°37'60.0"S |  | 13 Jun 1982 | Gentry | 36801 | BR | | A |
| *S. rosea* | CR | 10°21'00"N | 84°40'00"W | 800 m | 14 Feb 1994 | Haber | 11778 | F | | B |
| *S. rosea* | CR | 10°13'0.0"N | 84°35'10.0"S | 900 m |  | Azofeifa | 10 | MO | | A |
| *S. rosea* | CR | 10°17'00"N | 84°47'00"W | 950 m | 18 Jul 1996 | Krings | 104 | F | | B |
| *S. rosea* | CR | 10°12'52.99"N | 84°36'27.97"S |  |  | Herrera | 480 | MO | | A |
| *S. rosea* | CR | 10°13'00"N | 84°35'20"W | 800–1000 m | 27 Apr 1993 | Cano et al. | 48 | F | | B |
| *S. rosea* | CR | 10°18'N | 84°45'W | 900 m | 21 May 1987 | Haber & Bello | 7166 | WIS | | B |
| *S. rosea* | CR | 10°51'48.0"N | 85°19'34.2"S | 792 m |  | Zamora | 9320 | CR | | A |
| *S. rosea* | CR | 09°46'58"N | 83°45'20"W | 1000–1100 m | 19 Jan 1996 | Cascante et al. | 953 | F | | B |
| *S. rosea* | CR | 10°59'25.0"N | 85°25'40.0"S | 850 m |  | Moraga |  | INB | | A |
| *S. rosea* | CR | 10°47'2.0"N | 85°18'10.0"S | 1240 m |  | Rivera | 1213 | MO | | A |
| *S. rosea* | CR | 10°46'9.0"N | 85°17'25.0"S | 900 m |  | Rivera |  | INB | | A |
| *S. rosea* | CR | 9°27'20.0"N | 83°11'24.0"S | 1050 m |  | Santamaría |  | INB | | A |
| *S. rosea* | CR | 8°53'51.87"N | 82°46'4.5"S | 1400 |  | Alfaro |  | INB | | A |
| *S. rosea* | CR | 8°29'50.0"N | 83°28'55.0"S | 550 m |  | Herrera |  | INB | | A |
| *S. rosea* | CR | 9°18'0.0"N | 83°17'60.0"S | 800 m |  | Herrera | 3472 | CR | | A |
| *S. rosea* | CR | 8°53'24.0"N | 82°45'36.0"S | 1400 |  | Alfaro | 1090 | MO | | A |
| *S. rosea* | CR | 9°4'60.0"N | 83°4'10.0"S | 1250 m |  | Rodríguez |  | INB | | A |
| *S. rosea* | CR | 8°29'24.0"N | 83°28'48.0"S | 600 m |  | Herrera | 4730 | CR | | A |
| *S. rosea* | CR | 9°40'19.99"N | 84°9'45.0"S | 1000 m |  | Morales | 6840 | MO | | A |
| *S. rosea* | CR | 10°13'0.01"N | 84°35'10.0"S | 900 m |  | Zuñiga | 10 | B | | A |
| *S. rosea* | CR | 9°49'0.0"N | 84°28'50.0"S | 1200 m |  | Zúñiga |  | INB | | A |
| *S. rosea* | EC | 01°06'N | 78°14'W | 900–1100 m | 20 Apr 1993 | Méndez et al. | 397 | MO, WIS | | B |
| *S. rosea* | EC | 0°56'42.0"S | 78°58'19.2"S | 1025 m | 07 Feb 1982 | Dodson | 12225 | BR | | A |
| *S. rosea* | EC | 03°28'S | 78°15'W | 1350 m | 17 Jul 1993 | Gentry | 80018 | MO | | B |
| *S. rosea* | EC | 00°02'N | 77°33'W | 1390 m | 27 May 2000 | Guevara | s.n. | QCA | | B |
| *S. rosea* | EC | 01°25'S | 77°58'W | 1050 m | 03 Jul 1985 | Stein & Tucker | 3105 | NY | | B |
| *S. rosea* | EC | 0°12'1.0"N | 77°31'54.0"S | 1535 |  | Aguinda | 1297 | ECUAMZ, F, HUTPL | | A |
| *S. rosea* | EC | 0°12'1.0"N | 77°31'54.0"S |  |  | Aguinda | 1711 | F | | A |
| *S. rosea* | EC | 01°21'S | 78°20'W | 1400 m | 24 Jun 1987 | Palacios | 1646 | MO | | B |
| *S. rosea* | EC | 1°23'55.97"S | 78°23'10.0"S | 800 m |  | Ramírez | 19 | MO | | A |
| *S. rosea* | EC | 01°23'56"S | 78°23'10"W | 1800 m | 26 Nov 2000 | Neill & Canaday | 12931 | MO | | B |
| *S. rosea* | EC | 1°23'55.97"S | 78°23'10.0"S | 1800 m |  | Neill | 12931 | AAU | | A |
| *S. rosea* | EC | 1°23'60.0"S | 78°22'58.8"S | 1800 m |  | Balslev | 62464 | AU | | A |
| *S. rosea* | EC | 3°58'0.0"S | 79°4'0.0"S | 1900 m |  | Cabrera | 1918 | HUTPL | | A |
| *S. rosea* | EC | 3°47'12.0"S | 78°29'42.0"S | 1435 m |  | Neill | 16901 | AAU | |  |
| *S. rosea* | EC | 3°57'59.98"S | 79°3'59.98"S |  |  | Homeier | 379 | BIEL | | A |
| *S. rosea* | EC | 4°15'32.0"S | 78°41'4.0"S | 1620 m |  | Neill | 15468 | ECUAMZ | | A |
| *S. rosea* | EC | 3°46'17.0"S | 78°29'28.0"S | 1610 m |  | Neill | 16330 | QCNE | | A |
| *S. rosea* | EC | 3°46'23.0"S | 78°29'54.0"S | 1550 m |  | Neill | 16293 | QCNE | | A |
| *S. rosea* | EC | 4°8'36.0"S | 78°38'44.0"S | 970 m |  | Neil | 15855 | AUU, US | | A |
| *S. rosea* | EC | 04°07'S | 78°37'W | 1200–1300 m | 20 Oct 1991 | Palacios et al. | 8428 | MO, SCZ | | B |
| *S. rosea* | NI | 10°57'59.98"N | 84°19'59.99"S | 60 m |  | Rueda | 17980 | HULE | | A |
| *S. rosea* | PA | 8°37'59.99"N | 80°7'59.99"S | 1000–1200 m | 07 Feb 1947 | Allen & Allen | 4193 | G, MO | | B |
| *S. rosea* | PA | 8°37'23.99"N | 80°6'29.99"S | 1000 m |  | Allen | 2924 | MO | | A |
| *S. rosea* | PA | 8°3'30.0"N | 77°16'60.0"S | 850 m |  | Hammel | 16279 | MO | | A |
| *S. rosea* | PA | 8°7'60.0"N | 77°16'60.0"S | 1250–1450 m | 26 Jan 1975 | Gentry & Mori | 13911 | MO | | B |
| *S. rosea* | PA | 8°30'59.98"N | 81°6'59.98"S | 850 m |  | Scott | 4879 | MO | | A |
| *S. rosea* | PA | 8°28'55.99"N | 81°5'52.98"S | 762 m |  | Allen | 4413 | MO | | A |
| *S. rosea* | PA | 08°10'N | 81°15'W |  | Feb 1849 | Seeman | 1630 | B |  |  |
| *S. rosea* | PE | 05°47'S | 77°53'W | 1840–2020 m | 09 Jul 1999 | Sánchez-Vega et al. | 10009 | F | | B |
| *S. rosea* | PE | 5°59'4.0"S | 78°36'3.0"S | 1758 m |  | Perea | 2490 | AAU | | A |
| *S. rosea* | PE | 5°58'59.99"S | 79°12'0.0"S | 1500 m |  | Campos | 2962 | MO | | A |
| *S. rosea* | PE | 5°18'30.0"S | 78°44'1.0"S | 1550 m |  | Campos | 4195 | MO | | A |
| *S. rosea* | PE | 05°25'S | 78°53'W | 1780 m | 5 feb 1988 | Gentry et al. | 61212 | MO | | B |
| *S. rosea* | PE | 12°56'12"S | 72°47'03"W | 2000–2500 m | 25 Sep 2002 | Valenzuela et al. | 656 | F | | B |
| *S. rosea* | PE | 09°57'S | 75°37'W | 1000–1300 m | 19 Jun 1913 | Weberbauer | 6770 | F, G, USM | | B |
| *S. rosea* | PE | 11°15'S | 75°20'W |  | 05 Jun 1983 | Gentry et al. | 41511 | MO, USM | | B |
| *S. rosea* | PE | 5°49'59.99"S | 77°45'0.0"S | 1850 m |  | David | 4477 | MO | | A |
| *S. rosea* | PE | 05°50'S | 77°45'W | 1800–1900 m | 29 Jul 1983 | Smith | 4477 | MO, NY | | B |
| *S. rosea* | VE | 1°57'0.0"N | 66°58'0.0"S |  | 29 Apr 1974 | Morillo | 4159 | BR | | A |
| *S. rosea* | VE | 1°55'60.0"N | 67°1'60.0"S |  | 24 Apr 1974 | Morillo | 3905 | BR | | A |
| *S. rosea* | VE | 3°40'0.0"N | 65°45'0.0"S |  | 28/30 Jan y 6/8 de Feb de 1982 | Steyermark | 125647 | BR | | A |
| *S. rosea* | VE | 3°40'0.0"N | 66°49'60.0"S |  | 14/28 Feb 1978 | Huber | 1651 | BR | | A |
| *S. rosea* | VE | 3°40'0.0"N | 66°43'60.0"S |  | 28 Jun 1979 | Huber | 3890 | BR | | A |
| *S. rosea* | VE | 3°37'0.0"N | 67°25'60.0"S |  | 23 Feb 1979 | Huber | 3350 | BR | | A |
| *S. rosea* | VE | 5°50'60.0"N | 67°28'60.0"S |  | 17 Apr 1978 | Davidse | 15249 | BR | | A |
| *S. rosea* | VE | 4°47'60.0"N | 67°25'60.0"S |  | 16 Jul 1980 | Huber | 5340 | BR | | A |
| *S. rosea* | VE | 4°16'60.0"N | 67°16'60.0"S |  | 09 Mar 1980 | Huber | 5092 | BR | | A |
| *S. rosea* | VE | 4°10'0.0"N | 67°22'60.0"S |  | 21 Feb 1979 | Huber | 3295 | BR | | A |
| *S. rosea* | VE | 6°7'0.0"N | 63°22'0.0"S |  | 23 Mar 1985 | Huber | 10323 | BR | | A |
| *S. rosea* | VE | 6°31'0.0"N | 62°52'60.0"S |  | 29 Aug 1983 | Huber | 8151 | BR | | A |
| *S. rosea* | VE | 5°34'60.0"N | 61°30'0.0"S |  | 07 Mar 1983 | Huber | 7409 | BR | | A |
| *S. zapotillana* | EC | 3°52'22"S | 80°05'31"W | 300–900 m | Aug 1978 | Daly | 071 | NY | | C |
| *S. zapotillana* | EC | 4°8'22.93"S | 80°21'41.18"W | 566 m | 26 Nov 2024 | Armijos et al. | 3449 | HUTPL | | C |
| *S. zapotillana* | EC | 4°8'23.26"S | 80°21'41.07"W | 565 m | 26 Nov 2024 | Armijos et al. | 3450 | HUTPL | | C |
| *S. zapotillana* | EC | 4°9'40.61"S | 80°22'33.53"W | 461 m | 14 Jan 2025 | Armijos et al. | 3452 | HUTPL | | C |
| *S. zapotillana* | EC | 4°9'40.85"S | 80°22'33.68"W | 462 m | 14 Jan 2025 | Armijos et al. | 3453 | HUTPL | | C |
| *S. zapotillana* | EC | 3°59'57.66"S | 80°20'31.19"W | 183 m | 08 Jul 2025 | Armijos et al. | 3467 | HUTPL | | C |
| *S. zapotillana* | EC | 4°0'52.08"S | 80°19'15.52"W | 299 m | 08 Jul 2025 | Armijos et al. | 3468 | HUTPL | | C |
| *S. zapotillana* | EC | 4°0'8.23"S | 80°20'8.5"W | 198 m | 08 Jul 2025 | Armijos et al. | 3469 | HUTPL | | C |
| *S. zapotillana* | EC | 3°59'26.97"S | 80°22'51.1"W | 117 m | 08 Jul 2025 | Armijos et al. | 3470 | HUTPL | | C |
| *S. zapotillana* | EC | 4°4'55.47"S | 80°26'16.03"W | 507 m | 09 Jul 2025 | Armijos et al. | 3471 | HUTPL | | C |
| *S. zapotillana* | EC | 3°59'46.05"S | 80°21'10.77"W | 185 m | 08 Jul 2025 | Armijos et al. | 3472 | HUTPL | | C |
| *S. zapotillana* | EC | 3°59'46.42"S | 80°21'18.07"W | 181 m | 08 Jul 2025 | Armijos et al. | 3473 | HUTPL | | C |
| *S. zapotillana* | EC | 4°13'40.81"S | 80°18'32.34"W | 481 m | 13 Mar 2025 | Armijos et al. | 3454 | HUTPL | | C |
| *S. zapotillana* | EC | 4°13'41.47"S | 80°18'32.14"W | 480 m | 13 Mar 2025 | Armijos et al. | 3455 | HUTPL | | C |
| *S. zapotillana* | EC | 4°13'41.75"S | 80°18'31.95"W | 480 m | 13 Mar 2025 | Armijos et al. | 3456 | HUTPL | | C |
| *S. zapotillana* | EC | 4°13'42.54"S | 80°18'31.23"W | 479 m | 13 Mar 2025 | Armijos et al. | 3457 | HUTPL | | C |
| *S. zapotillana* | EC | 4°13'42.54"S | 80°18'31.23"W | 479 m | 16 May 2025 | Armijos et al. | 3443 | paratype: HUTPL | | C |
| *S. zapotillana* | EC | 4°13'43.13"S | 80°17'51.92"W | 468 M | 28 May 2025 | Armijos et al. | 3465 | HUTPL | | C |
| *S. zapotillana* | EC | 4°13'44.38"S | 80°17'51.13"W | 473 m | 28 May 2025 | Armijos et al. | 3466 | HUTPL | | C |
| *S. zapotillana* | EC | 4°13'45.7"S | 80°17'53.47"W | 468 M | 27 Nov 2024 | Armijos et al. | 3462 | HUTPL | | C |
| *S. zapotillana* | EC | 4°13'46.43"S | 80°17'53.23"W | 469 m | 27 Nov 2024 | Armijos et al. | 3463 | HUTPL | | C |
| *S. zapotillana* | EC | 4°13'46.6"S | 80°17'53.24"W | 468 m | 27 Nov 2024 | Armijos et al. | 3464 | HUTPL | | C |
| *S. zapotillana* | EC | 4°7'22.62"S | 80°24'31.28"W | 453 m | 16 May 2025 | Armijos et al. | 3458 | HUTPL | | C |
| *S. zapotillana* | EC | 4°7'25.85"S | 80°24'26.82"W | 463 m | 16 May 2025 | Armijos et al. | 3459 | HUTPL | | C |
| *S. zapotillana* | EC | 4°7'26.33"S | 80°24'26.87"W | 464 m | 16 May 2025 | Armijos et al. | 3448 | paratype: HUTPL | | C |
| *S. zapotillana* | EC | 4°7'26.44"S | 80°24'26.67"W | 469 m | 16 May 2025 | Armijos et al. | 3460 | HUTPL | | C |
| *S. zapotillana* | EC | 4°3'26.59"S | 80°18'23.26"W | 380 m | 09 Jul 2025 | Armijos et al. | 3474 | HUTPL | | C |
| *S. zapotillana* | EC | 4°7'49.11"S | 80°25'30.87"W | 496 m | 16 May 2025 | Armijos et al. | 3444 | paratype: HUTPL | | C |
| *S. zapotillana* | EC | 4°7'49.12"S | 80°25'30.9"W | 496 m | 16 May 2025 | Armijos et al. | 3446 | paratype: HUTPL | | C |
| *S. zapotillana* | EC | 4°7'50.99"S | 80°25'30.82"W | 499 m | 26 Nov 2024 | Armijos et al. | 3451 | HUTPL | | C |
| *S. zapotillana* | EC | 4°7'51.02"S | 80°25'30.51"W | 501 m | 27 May 2025 | Armijos et al. | 3461 | HUTPL | | C |
| *S. zapotillana* | EC | 4°8'30.52"S | 80°25'32.87"W | 473 m | 15 Jan 2025 | Armijos et al. | 3445 | holotype: HUTPL | | C |
| *S. zapotillana* | EC | 4°7'25.83"S | 80°24'26.98"W | 463 m | 16 May 2025 | Armijos et al. | 3447 | paratype: HUTPL | | C |
