# Supplementary material for: A new tree species from seasonally dry tropical forest in southern Ecuador, Spirotheca zapotillana sp. nov. (Malvaceae), resolves a putatively disjunct distribution
Source: PhytoKeys. 2025 Oct 31;265:181–92. doi: 10.3897/phytokeys.265.162409 (PMC12595510; doi:10.3897/phytokeys.265.162409)
Supplement: Supplementary material 2 — Data matrix for the phylogenetic analysis of Spirotheca [file phytokeys-265-181_article-162409__-s002.docx]

**Supplementary material 2.** Data matrix for the phylogenetic analysis of *Spirotheca*. Morphological characters utilized in the analysis were obtained from protologues (Cuatrecasas 1954; Fernández-Alonso 2001; Carvalho-Sobrinho et al. 2012) and a monographic study (Gibbs et al. 2006). Qualitative and quantitative traits were transformed into multistate characters.

| **No** | **Character** | **Character states** | ***S. awadendrom*** | ***S. elegans*** | ***S. mahechae*** | ***S. michaeli*** | ***S. rivieri*** | ***S. rosea*** | ***S. zapotillana*** |
| --- | --- | --- | --- | --- | --- | --- | --- | --- | --- |
| 1 | Habit | [0] tree; [1] strangler tree | 0 | 1 | 1 | 1 | 0 | 1 | 0 |
| 2 | Height (m) | [0] up to 20; [1] up to 35; [2] >35 | 2 | 0 | 0 |  | 1 | 2 | 0 |
| 3 | Grayish strips on trunk | [0] absent; [1] present |  | 1 |  |  |  |  | 1 |
| 4 | Prickles on branches and trunk | [0] absent; [1] present | 1 | 1 |  |  |  |  | 1 |
| 5 | Length of prickles (mm) | [0] up to 7; [1] to 14; [2] >14 | 0 | 2 |  |  |  |  | 2 |
| 6 | Width of prickles (mm) | [0] up to 8; [1] > 8 | 0 |  |  |  |  |  | 1 |
| 7 | Number of leaftles | [0] to 5; [1] >5 | 1 | 0 | 1 | 1 | 1 | 1 | 1 |
| 8 | Length of petiole (mm) | [0] to 60; [1] to 100; [2] >100 | 2 | 1 | 1 | 0 | 0 | 2 | 1 |
| 9 | Width of petiole (mm) | [0] to 2; [1] > 2 | 1 |  | 0 |  |  |  | 0 |
| 10 | Shape of petiole base | [0] non-pulvinate; [1] pulvinate | 1 | 1 |  | 1 |  |  | 1 |
| 11 | Petiole indumentum | [0] absent; [1] present | 1 |  |  | 0 |  |  | 0 |
| 12 | Shape of leaflets | [0] oblong; [1] elliptic |  |  |  |  |  |  |  |
| 13 | Adaxial leaf surface indumentum | [0] absent; [1] present | 0 | 0 | 0 | 0 | 0 | 0 | 0 |
| 14 | Abaxial leaf surface indumentum | [0] absent; [1] present | 1 | 0 |  | 1 | 0 | 1 | 0 |
| 15 | Petiolule | [0] absent; [1] to 3; [2] to 6; [3] >6 | 3 | 0 | 3 |  | 1 | 2 | 2 |
| 16 | Shape of leaflet apex | [0] acute; [1] obtuse | 0 | 1 | 1 | 0 | 1 | 0 | 0 |
| 17 | Length of leaflet (mm) | [0] to 60; [1] to 100; [2] >100 | 2 | 0 | 0 | 2 | 1 | 2 | 1 |
| 18 | Width of leaflet (mm) | [0] to 24; [1] to 40; [2] >40 | 2 | 0 | 0 | 2 | 1 | 1 | 1 |
| 19 | Leaflet texture | [0] coriaceous; [1] membranaceous | 0 | 1 | 0 | 0 |  |  | 1 |
| 20 | Inflorescence | [0] flowers isolate; [1] cymes; [2] fascicles | 0 | 2 | 0 | 0 | 0 | 0 | 1 |
| 21 | Number of flowers | [0] to 3; [1] >3 |  | 1 |  |  |  |  | 1 |
| 22 | Orientation of flower | [0] erect; [1] downwards |  | 1 | 0 |  |  |  | 0 |
| 23 | Length of pedicel (mm) | [0] to 15; [1] to 20; [2] >20 | 0 | 2 |  |  | 2 | 1 | 2 |
| 24 | Glands on receptacle | [0] absent; [1] present |  | 1 |  |  |  |  | 1 |
| 25 | Shape of calyx | [0] cupuliform; [1] obconic | 0 | 0 | 1 | 0 | 0 | 0 | 0 |
| 26 | Calyx indument | [0] absent; [1] present | 1 | 0 | 1 | 0 | 0 | 0 | 0 |
| 27 | Margin of calyx | [0] truncate; [1] lobed; [2] apiculate | 1 | 2 | 1 | 1 | 0 | 1 | 2 |
| 28 | Length of calyx (mm) | [0] to 9; [1] to 14; [2] >14 | 2 | 0 | 2 |  | 0 | 2 | 0 |
| 29 | Width of calyx (mm) | [0] to 11; [1] to 16; [2] >16 | 2 | 0 | 2 |  |  |  | 1 |
| 30 | Color of petals | [0] white; [1] red; [2] pink | 0 | 0 | 2 | 1 | 1 | 1 | 1 |
| 31 | Shape of petals | [0] lanceolate; [1] oblong | 0 | 1 | 0 | 1 | 1 | 1 | 1 |
| 32 | Longitudinal symmetry of petals | [0] asymmetry; [1] symmetry |  | 0 |  |  |  |  | 0 |
| 33 | Apex shape of petals | [0] acute; [1] obtuse | 1 |  | 0 | 1 |  |  | 0 |
| 34 | External indumentum of petals | [0] absent; [1] present | 1 | 1 | 1 | 1 | 1 | 1 | 1 |
| 35 | Internal indumentum of petals | [0] absent; [1] present | 1 | 1 | 1 | 1 | 1 | 1 | 1 |
| 36 | Length of petals (mm) | [0] to 66; [1] to 93; [2] >93 | 1 | 0 | 1 | 2 | 0 | 2 | 0 |
| 37 | Width of petals (mm) | [0] to 11; [1] to 18; [2] >18 | 1 | 0 |  | 0 | 0 | 2 | 1 |
| 38 | Type of staminal articulation | [0] non-articulate; [1] simple-articulate; [2] doubly articulate; [3] non-articulate or articulate | 1 | 2 | 0 | 0 | 3 | 1 | 0 |
| 39 | Indument of staminal tube at base | [0] absent; [1] present | 1 | 1 | 1 | 1 | 1 | 1 | 1 |
| 40 | Indumentum of staminal tube at apex | [0] absent; [1] present | 0 | 0 | 1 | 0 | 1 | 1 | 1 |
| 41 | Length of staminal tube (mm) | [0] to 24; [1] to 37; [2] >37 | 2 | 1 | 2 | 1 | 2 | 2 | 1 |
| 42 | Color of filaments | [0] red; [1] pink |  | 1 | 1 |  |  | 0 | 0 |
| 43 | Length of filaments (mm) | [0] to 17; [1] to 28; [2] >28 |  | 0 | 1 | 2 | 0 | 1 | 0 |
| 44 | Length of upper thecae (mm) | [0] to 7; [1] to 12; [2] >12 |  | 0 |  | 2 | 1 | 2 | 1 |
| 45 | Length of lower thecae (mm) | [0] to 9; [1] to 16; [2] >16 |  | 0 |  | 2 | 1 | 2 | 0 |
| 46 | Shape of ovary | [0] conic; [1] oblong |  | 0 |  | 1 |  | 0 | 0 |
| 47 | Color of style | [0] red; [1] pink |  | 1 |  |  |  | 0 | 0 |
| 48 | Indumentum of style | [0] absent; [1] present |  |  |  | 0 | 1 | 1 | 0 |
| 49 | Length of style (mm) | [0] to 16; [1] to 18; [2] >18 |  | 0 |  |  |  |  | 2 |
| 50 | Orientation of Style | [0] erect; [1] declinate |  | 1 |  | 0 |  |  | 1 |
| 51 | Shape of stigma | [0] lobed; [1] branched |  | 0 |  |  |  | 0 | 1 |
| 52 | Shape of fruits | [0] elliptic; [1] oblong; [2] ovoide | 0 | 2 |  |  | 0 |  | 1 |
| 53 | Length of fruit (mm) | [0] to 40; [1] to 110; [2] >110 | 1 | 1 |  |  | 1 | 2 | 1 |
| 54 | Width of fruit (mm) | [0] to 27; [1] to 41; [2] >41 | 1 | 1 |  |  | 2 |  | 0 |
| 55 | Color of kapok | [0] white; [1] brown | 1 | 0 |  |  |  |  | 1 |
| 56 | Shape of the seeds | [0] reniform; [1] pyriform | 0 | 1 |  |  |  |  | 0 |
