# Supplementary material for: A new tree species from seasonally dry tropical forest in southern Ecuador, Spirotheca zapotillana sp. nov. (Malvaceae), resolves a putatively disjunct distribution
Source: PhytoKeys. 2025 Oct 31;265:181–92. doi: 10.3897/phytokeys.265.162409 (PMC12595510; doi:10.3897/phytokeys.265.162409)
Supplement: Supplementary material 3 — Supplementary figures [file phytokeys-265-181_article-162409__-s003.pdf]

HERBARIO

15354

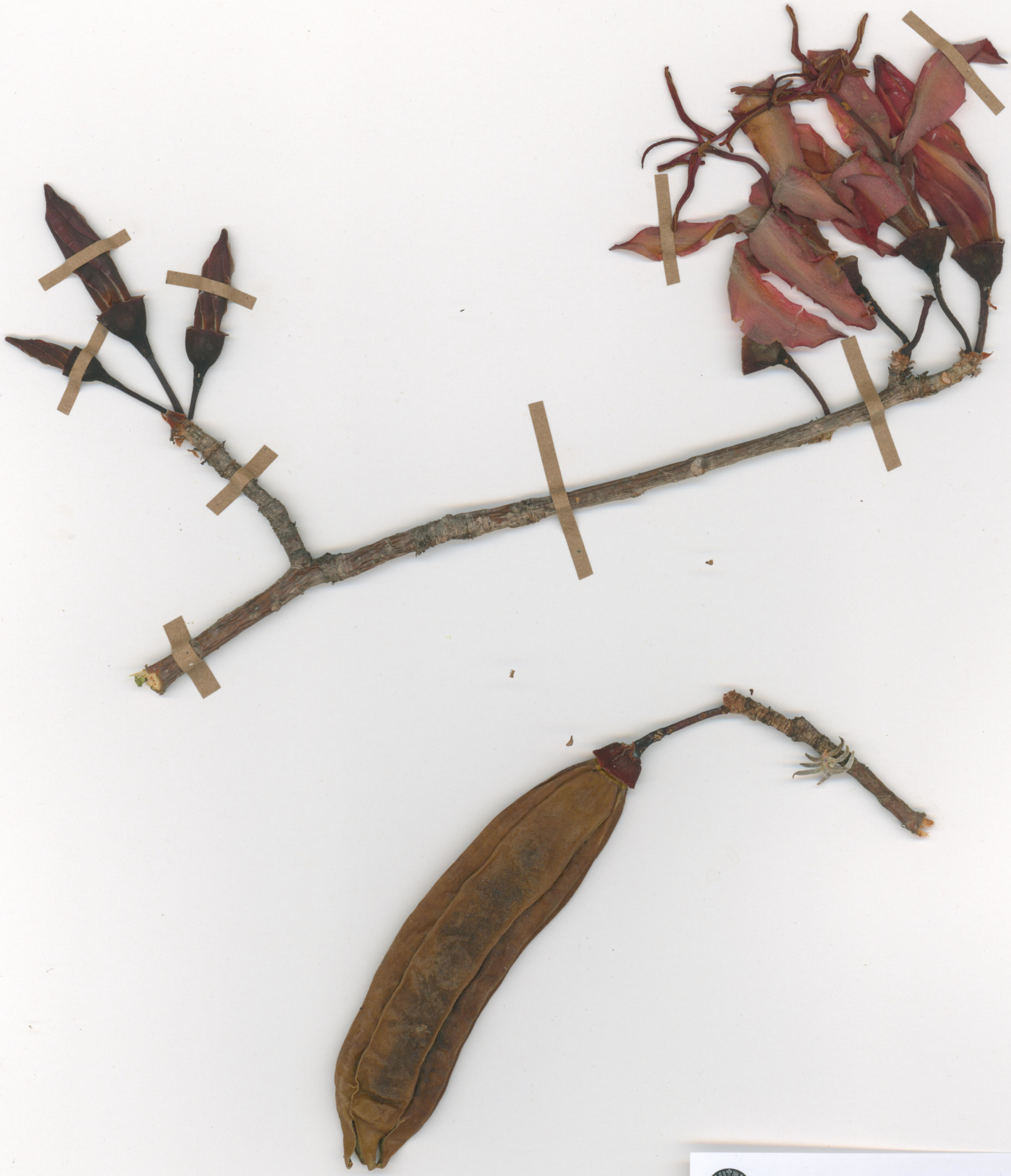

**FLORA DE ECUADOR**
**UNIVERSIDAD TECNICA PARTICULAR DE LOJA**

**Malvaceae**

*Spirotheca* sp.nov.

Det.: J.L. Armijos–Barros, HUTPL, 2025–04–25

Loja. Zapotillo. Parroquia Mangahurco (cod. 6794). Bosque seco. 4°8'30.52S 80°25'32.87W. 473 m

Árbol de 11.7 m. Tronco gris cubierto por aguijones. Hojas generalmente con 5 folíolos. Flores rojizo–brillantes colectadas en agosto de 2025. Frutos colectados en septiembre de 2025

Col.: J.L. Armijos–Barros et al. 3445. 2025–01–15

**Estudio de diversidad taxonómica y genética de plantas y hongos del Ecuador. MAATE–CMARG–2025–0064**

HUTPL
HERBARIO

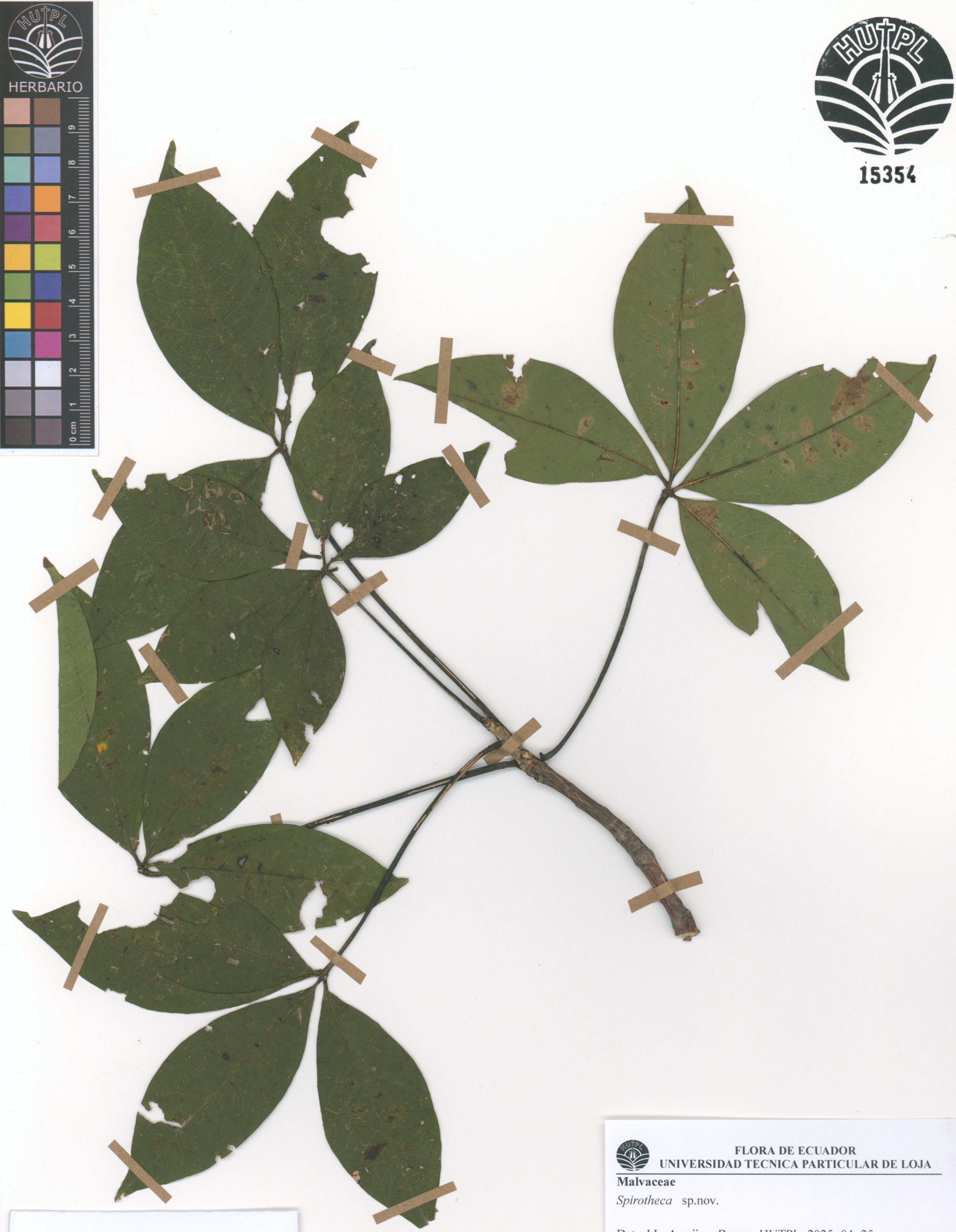

15354

**FLORA DE ECUADOR**
**UNIVERSIDAD TECNICA PARTICULAR DE LOJA**

**Malvaceae**

*Spirotheca* sp.nov.

Det.: J.L. Armijos−Barros, HUTPL, 2025−04−25

Loja. Zapotillo. Parroquia Mangahurco (cod. 6794). Bosque seco. 4°8'30.52S 80°25'32.87W. 473 m

Árbol de 11.7 m. Tronco gris cubierto por aguijones. Hojas generalmente con 5 folíolos. Flores rojizo−brillantes colectadas en agosto de 2025. Frutos colectados en septiembre de 2025

Col.: J.L. Armijos−Barros et al. 3445. 2025−01−15

**Estudio de diversidad taxonómica y genética de plantas y hongos del Ecuador. MAATE−CMARG−2025−0064**
